# Using interactive data visualization to facilitate user selection and comparison of risk of bias tools for observational studies of exposures

**DOI:** 10.1016/j.envint.2020.105806

**Published:** 2020-06-05

**Authors:** Kyla W. Taylor, Zhicheng Wang, Vickie R. Walker, Andrew A. Rooney, Lisa A. Bero

**Affiliations:** a Division of the National Toxicology Program, National Institute of Environmental Health Sciences, NIH, DHHS, NC, USA; b Charles Perkins Centre, The University of Sydney, Sydney, Australia; c Sydney Pharmacy School, Faculty of Medicine and Health, The University of Sydney, Sydney, Australia

## 1. Introduction

The evaluation of risk of bias of individual studies is a critical step in the systematic review process (Bero et al., 2018). Risk of bias is commonly defined as characteristics of the design, conduct or analysis of a study related to systematic error, or deviation from the truth, in results that assess the risk of a study over- or underestimating outcome effects (Boutron et al., 2019; Viswanathan et al., 2013) (see Box 1). Although significant progress has been made in developing tools to assess risk of bias for a variety of study types (Higgins et al., 2011; Juni et al., 2001; Katrak et al., 2004; Sanderson et al., 2007; Sterne et al., 2016), there is no consensus on optimal tools for assessing risk of bias in observational studies (Bero et al., 2018).

In the absence of clear consensus, there is a need for an easy and accessible way for systematic reviewers and guideline developers to compare different tools to find ones that are fit for purpose. Interactive data visualization tables that can facilitate this type of comparison, but requires no expertise or special training, would be a valuable resource. In order to meet this need, we (the National Toxicology Program and the Charles Perkins Centre at the University of Sydney) partnered with the National Health and Medical Research Council (NHMRC), Research Translation Department, World Health Organization, Guidelines Secretariat to (1) conduct a systematic review to identify, describe and evaluate tools designed to assess risk of bias in observational studies of exposure and (2) create corresponding interactive visualization tables that would enable users to easily find and compare available tools and different components within tools. The purpose of this editorial is to introduce readers to these interactive visualization tables, called the *Tableau* risk of bias tool finder, and guide systematic reviewers on how it can be utilized to find a risk of bias tool that is best suited to their needs.

## 2. Systematic review of risk of bias tools for observational studies of exposures

We conducted a systematic review of risk-of-bias tools for observational studies of exposures (Wang et al., 2018) that built on previously published reviews of tools (Deeks et al., 2003; Rooney et al., 2016; Sanderson et al., 2007; Shamliyan et al., 2010). The Wang et al. (2018) review was published as a chapter in an online resource, the NHMRC’s Guidelines for Guidelines (https://www.nhmrc.gov.au/guidelinesforguidelines), a collection of peer reviewed modules that form a handbook covering the aspects of planning, development, review, implementation and updating of guidelines. Before being published online, each chapter is released in draft format for public comment and peer reviewed.

Readers may find a more detailed description of the methods used in Wang et al. (2018) here: https://www.nhmrc.gov.au/guidelinesforguidelines/assessing-risk-bias/tools-assess-risk-bias. In brief, exposure was defined as any exposure that is not controlled by the investigator; this could include exposures to nutrients, chemicals, and biological or physical stressors (e.g., air pollution or physical activity). We identified sixty-two tools that assess risk of bias in observational studies, extracted key information about the content of each tool, including specific questions and domains related to methodological quality or risk of bias (Wang et al., 2018). Questions related to risk of bias were grouped into 17 topics under 9 risk-of-bias domains: selection, exposure, outcome assessment, confounding, loss to follow-up, analysis, selective reporting, conflicts of interest, and other. The 9 domains were derived from the Cochrane risk of bias tool (Higgins and Green, 2011), the Navigation Guide (Woodruff and Sutton, 2014) and the National Research Council Review of the Environmental Protection Agency’s Integrated Risk Information Systems (IRIS) process (National Research Council, 2014).

To enable easy and effective comparison of the tools and questions within the tools, we organized and presented results in a tabular format using a novel method based on the emerging practices of evidence mapping, a form of evidence synthesis aimed at categorizing studies and study details by key concepts for addressing a research question. Using *Tableau*, a free and open source software, we developed interactive data visualization tables to allow users to explore features of the tools based on key concepts such as risk of bias domains. The interactive features of *Tableau* (Seattle, Washington, United States; https://www.tableau.com/) visualization software, have received growing attention in developing evidence maps (Wolffe et al., 2019). *Tableau* evidence maps, which include data interactive visualization tables, replace static text tables and leverage the categorical and quantitative information to assist users in comparing, contrasting, sorting, filtering, and summarizing the data, allowing users to explore collected information and come to their own conclusions.

## 3. Overview of the *Tableau* risk of bias tool finder

The interactive *Tableau* risk of bias tool finder (https://ntp.niehs.nih.gov/go/ohat_tools) describes each risk of bias tool in a format that allows users to critically examine and compare all 62 tools by characteristics of their development or usability and key concepts, including risk of bias domains and individual questions in the tools that address these domains (Table 1). The *Tableau* risk of bias tool finder would be useful in the field of evidence-based medicine, for example, as it can be used to find and compare tools by filtering them based on the research area of interest (e.g., surgery) and the design of the studies included in a review (e.g., cohort or case-control) to choose a tool that is the best fit for a specific evaluation.

Below we have created users guide to help users navigate the *Tableau* risk of bias tool finder. The purpose of this guide is to walk users through how to find and compare different tools based on the domains and questions these tools contain. The guide does not offer guidance on how users should implement an individual tool while conducting a systematic review. Many (but not all) of the tools provide their own guidance for how to use or apply the tool; however, the level and completeness of guidance does vary between tools. The National Toxicology Program’s OHAT tool (NTP, 2014), for example, provides detailed guidance whereas the Navigation Guide tool (Woodruff and Sutton, 2014) provides less guidance but has individual protocols and case-studies for examples of how the tool can be applied. In some cases, like in ROBINS-E (Morgan et al., 2017), for example, the user must define an ideal study to which they can compare the study being assessed. Therefore, it is important that users look to the tool-specific guidance for instructions on how the tools are meant to be used. It is also important to note that, depending on their specific topic or need, the user may need to modify or refine the tool.

## 4. User’s guide to the *Tableau* risk of bias tool finder

After clicking on the *Tableau* link, the user is taken to the “ReadMe” landing page. There are four tabs to the right of the “ReadMe” tab: “Information”, “Tool by Domain Name”, “Domain-Tool-Study Design”, and “Tools by Research Area”.

**Fig. 1.**
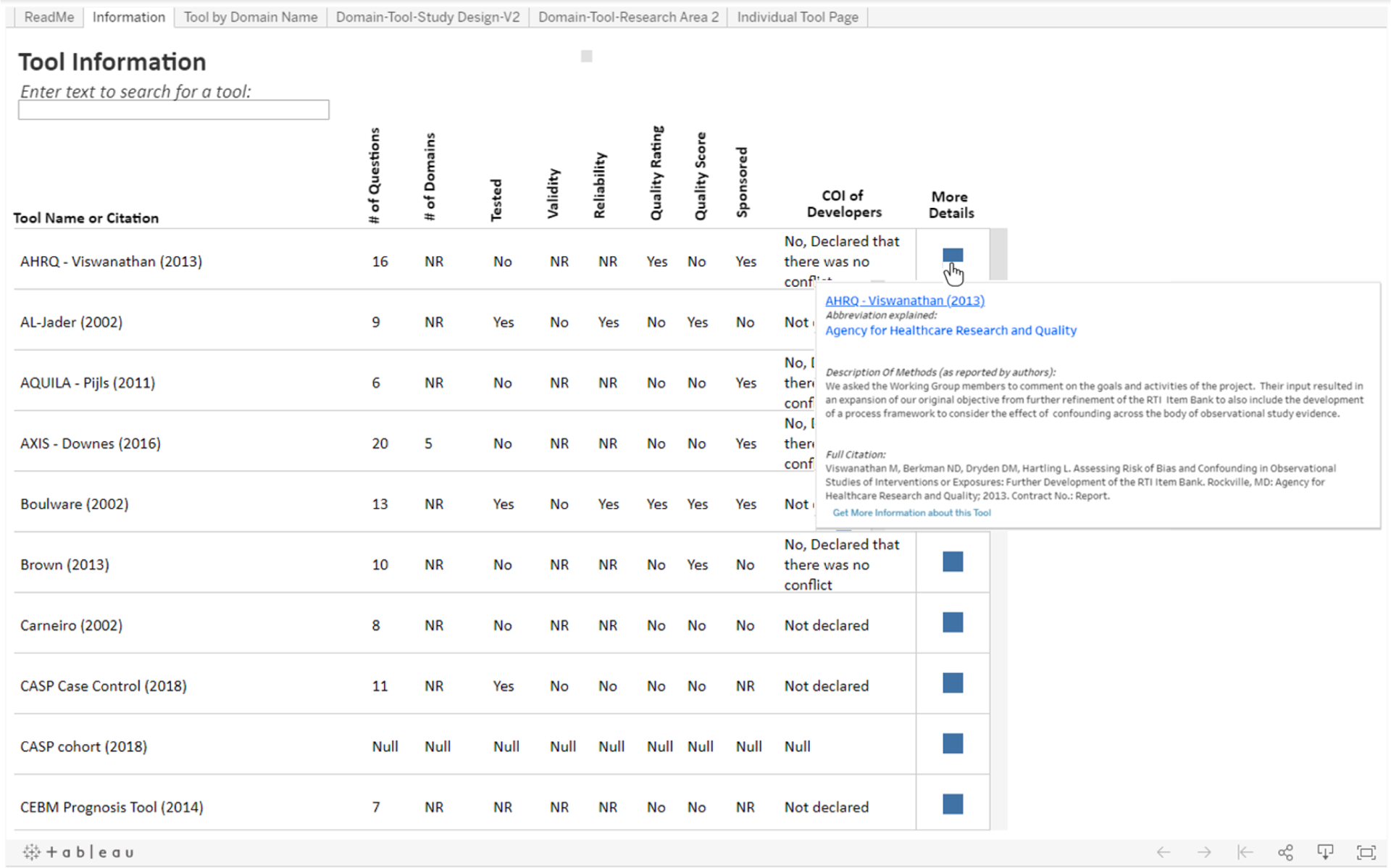
Summary information on characteristics and data on the development of the first ten tools, sorted alphabetically in the *Tableau* risk of bias tool finder. COI: Conflict of interest. NR: Not reported.

The **“Information”** tab (Fig. 1) displays summary information on tool characteristics and development for all 62 tools. This includes the number of domains and questions for each individual tool, if a quality score or rating is used, if the tool was tested for validity or reliability (as reported by the developers of the tool), if the tool’s development was sponsored and if the developers of the tool declare any conflicts of interest. A user of the *Tableau* risk of bias tool finder can use this information to quickly compare the characteristics of all available tools. If a user is interested in a specific tool, they can use the search box located on the upper left side of the page to find and display the information related to that tool. The user can also hover their mouse over or click on the blue box under the “More details” column to see the full name of the tool, the full citation of the tool and, if available, a description of the methods used in the development of each tool. The user can also click on a the “Get more information about this tool” link within the blue box to see the domain names, topics, and exact questions from that specific tool.

**Fig. 2.**
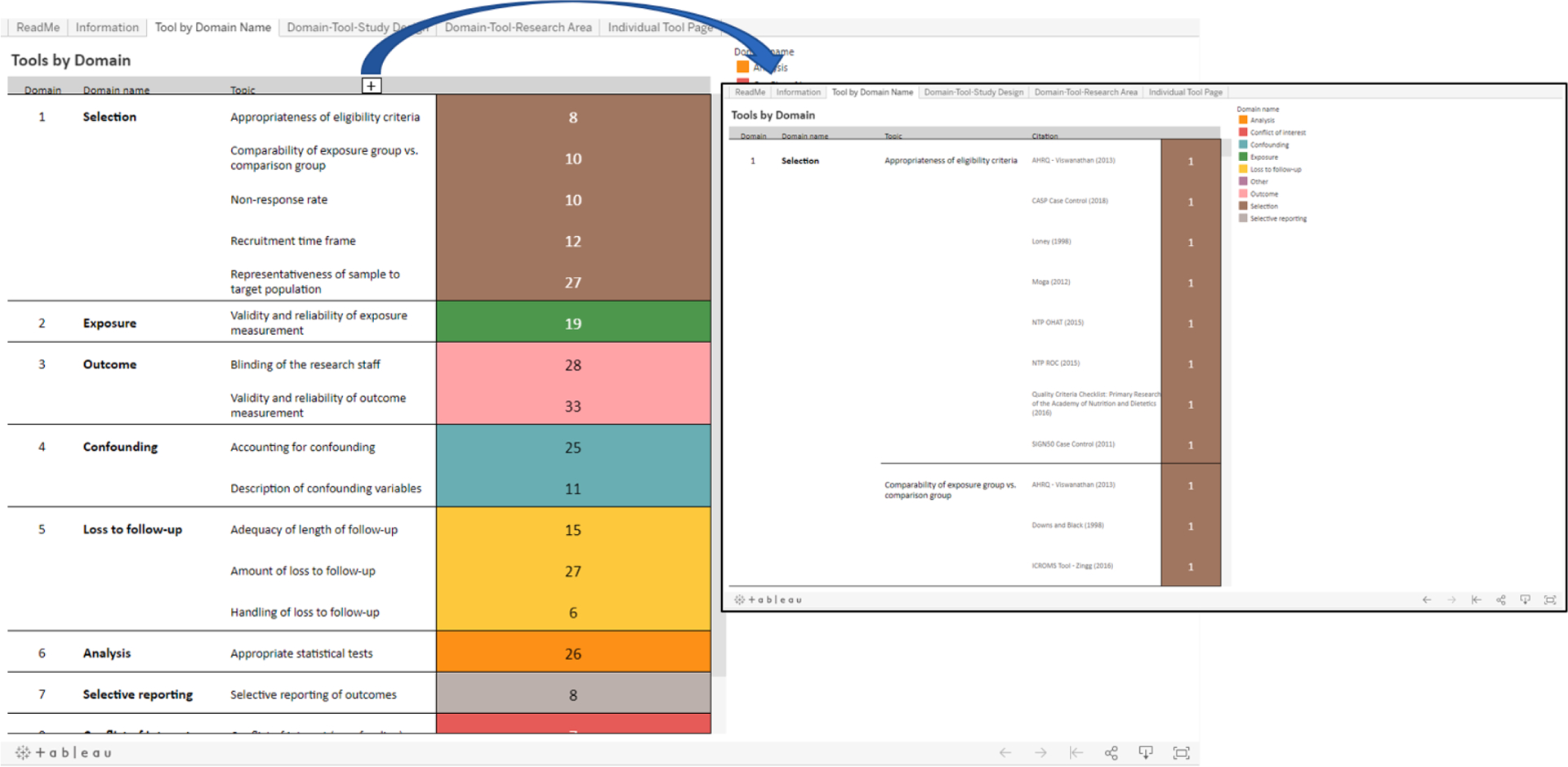
Information in the tab “Tools by Domain” summarizes the domains and topics that were related to risk of bias. [+] appears when hovering over the column labeled “topic”; clicking on this symbol will expand the topic column to display the specific tools that contain questions related to each topic. NR: Not reported. COI: Conflict of interest.

The **“Tool by Domain Name”** tab provides a table displaying each of the 17 topics grouped under 9 risk of bias domains: selection, exposure, outcome assessment, confounding, loss to follow-up, analysis, selective reporting, conflicts of interest and other. The numbers in the far right column correspond to how many individual tools contain a question that is relevant to each topic (Fig. 2). If a user is interested in knowing how different tools assessed risk of bias due to selection, for example, they will see that there are five topics grouped under the selection domain. If the user wants more information on the selection bias domain topic “Appropriateness of eligibility criteria”, they will see that there are 8 tools that assess that specific topic. The user can expand the topic column to view the names of the individual tools that contain questions related to this topic (Fig. 2). Once expanded, the user can hover their mouse over the number “1”, that is directly the right of a tool’s citation, and the exact question from the tool that is related to that topic will pop up. By doing this, the user can directly compare questions from different tools that address similar risk of bias issues. For example, by hovering their mouse, the user will see that the Agency for Healthcare Research and Quality (AHRQ) tool (Viswanathan et al., 2013) addresses “Appropriateness of eligibility criteria” by asking “Do the inclusion/exclusion criteria vary across the comparison groups of the study?” while NTP’s Office of Health Assessment and Translation (OHAT) tool (NTP, 2014) asks about this same topic with the question “Did selection of participants result in appropriate comparison groups”.

As can be examined in the “Tools by Domain Name” tab in *Tableau*, there was considerable variability between tools in the questions used to address the same risk of bias concept. For example, questions about the accuracy of outcome measurement included: “Are objective, suitable and standard criteria used for measurement of the health outcomes?” (Loney et al., 1998), “Was the outcome accurately measured to minimize bias?” (CASP, 2018) and “Were the risk factors and outcome variables measured correctly using instruments/measurements that had been trialed, piloted or published previously?” (Downes et al., 2016). Although these questions all assess the same concept (accuracy of outcome measurement), the tool a user picks may depend on their interpretation of how questions are worded, how clear questions are and whether they are applicable to the user’s specific evaluation.

The **“Domain-Tool-Study Design”** tab allows the user to filter domains, topics and individual questions by the study design for which different tools were developed to assess. To give an example of how a user might navigate the information in this tab, we will walk readers through a situation where a researcher wants to find and compare risk of bias tools that might be appropriate for a risk of bias assessment in a systematic review on case-control studies of environmental exposure. The researcher is also interested in how tools that are potentially relevant to their review address issues related to loss to follow-up.

The researcher may start in this tab if they want find tools that were designed to assess case-control studies. In the table “Study design for which the tool was used”, they will see that there are 36 tools that were designed to assess case-control studies. To filter based on the case-control study design, they can click on “Case Control” (or the number “36”) and the “List of relevant tools based on your selections” will update and display all 36 tools that were designed to assess case-control studies (Fig. 3a).

The researcher is also interested in how each tool that was designed to evaluates case-control studies assesses loss to follow-up. On the left side of the page, in the Domain table, next to the “Loss to follow-up” domain, the researcher will see that 10 tools have a question related to the topic “Adequacy of length of follow-up”, 16 tools have a question related to the topic “Amount of loss to follow-up” and 5 tools have a question related to the topic “Handling of loss to follow-up” (Fig. 3a). If the researcher wants to see which 16 tools contain a question related to the second topic, they can click on “Amount of loss to follow-up” and the “List of relevant tools based on your selections” will filter and display the names of the 16 tools (Fig. 3b).

The researcher can also compare the specific questions related to “Amount of loss to follow-up” from each tool by using the “Questions” table on the bottom right of the page. Because the researcher has already filtered on tools that assess case-control study designs and contain questions related to “Amount of loss to follow-up”, this table is already filtered to display the relevant tool names and corresponding question from each tool (Fig. 3b). The researcher can now easily compare the exact questions related to “Amount of loss to follow up” from each tool.

The final tab, **“Tools by Research Area”** displays the same type of interactive table as the “Domain-Tool-Study Design” tab but with additional information on the research area for which the tool was originally designed. Several tools included in Wang et al. (2018) were developed for a specific clinical or public health research area. For example, some of the tools were developed to assess research areas such as dentistry (Macfarlane et al., 2001), nutrition (Ohadike et al., 2016) and surgery (Manterola et al., 2006). We can return to the example of the researcher looking for tools to conduct a risk of bias assessment on case-control studies of environmental exposure. As a reminder, this researcher is also interested in how potentially relevant tools address issues related to loss to follow-up. The researcher now wants to examine tools that were designed to assess environmental exposures. Once in the “Tools by Research Area” tab, under the table “Research area for which the tool was developed to assess”, they will see that there are four tools designed to assess environmental exposures. If they click on the word “Environmental” (or the number “4”), all of the tables in this tab will automatically filter to only show information related to these four tools (NTP, 2015a, b; Roth and Wilks, 2014; Woodruff and Sutton, 2014) (Fig. 4a).

This researcher is also interested in how each tool that assesses environmental exposures approaches loss to follow-up. In the “Domain” table, next to the “Loss to follow-up” domain, the researcher will see that three tools have a question related to the topic “Amount of loss to follow-up” and one tool has a question related to the topic “Handling of loss to follow-up” (Fig. 4a). If the researcher wants to know which three tools have questions related to the former topic, they can click on the topic “Amount of loss to follow-up”. Upon doing so, the “List of relevant tools based on your selections” will filter and display the names of the three tools (NTP, 2015a, b; Woodruff and Sutton, 2014) (Fig. 4b).

To compare the specific questions related to “Amount of loss to follow-up” from each of these three tools, the researcher can look at the “Questions” table on the bottom right of the page. This table is sorted by study design and tool name. Because the researcher previously clicked on “Amount of loss to follow-up”, the table is already displaying the three tools that have questions related to “Amount of loss to follow-up”, sorted by study design. The researcher can use this table to compare the exact questions related to “Amount of loss to follow up” from each tool. They will see that the NTP OHAT tool addresses this topic with the question “Were outcome data complete without attrition or exclusion from analysis (detailed definitions of low and high risk of bias listed)” (NTP, 2015b), NTP RoC addresses it with the question “Is there a concern that follow-up was incomplete?” (NTP, 2015a), and that the Navigation Guide doesn’t have a specific question but addresses this topic with the domain “Incomplete outcome data” (Woodruff and Sutton, 2014) (Fig. 4b).

The *Tableau* risk of bias tool finder is designed to assist systematic reviewers or guideline developers unfamiliar with the different risk of bias tools that are available and to provide insight to experienced methodologists. The interactive nature of the *Tableau* risk of bias tool finder allows users to explore numerous components of risk of bias tools and to focus on what might be most informative to their project and background. To better illustrate the tool, we have provided an example of a hypothetical user: This user is about to conduct a review of cardiovascular effects of nutrition. The reviewer has several questions that the *Tableau* risk of bias tool finder can help answer, such as: What tools are available? (answer: 62 tools can be explored through in the tool). How many tools have been designed to address my research topic of interest (e.g., nutrition)? (answer: 2 tools were developed to address nutrition). What are the most common domains included in risk of bias tools? (answer: selection and outcome). Are both the selection and outcome domains included in the both tools developed to assess nutrition? (answer: no, only one of these two tools addresses both selection and outcome). My review consists of both case-control and cohort studies, can both tools assess both study designs? (answer: yes, both tools can assess case-control, case-series, cohort, and cross-sectional studies). Which tool is the best off the shelf for my cardiovascular research question? (answer: this depends on additional considerations for the researcher and their review team). After using the *Tableau* risk of bias tool finder, the hypothetical user may also choose to use one of the two tools or modify another tool to best address their needs.

## 5. Conclusion

The *Tableau* risk of bias tool finder provides a clear and interactive cross-walk across existing risk of bias tools that has not previously been available and has the potential to support new insights for researchers considering how to evaluate observational studies. By allowing users to filter tools by components of interest (e.g., study design, research area, risk of bias domain), the user can easily examine and compare the 62 tools and select a tool that is most appropriate to their needs. By providing information on available tools in a user-friendly format, the *Tableau* risk of bias tool finder can be used to promote a better understanding of these tools, facilitate the utilization of better evidence in evaluations and thereby improve the quality of conclusions in systematic reviews and the recommendations in guidelines.

## Figures and Tables

**Fig. 3a.**
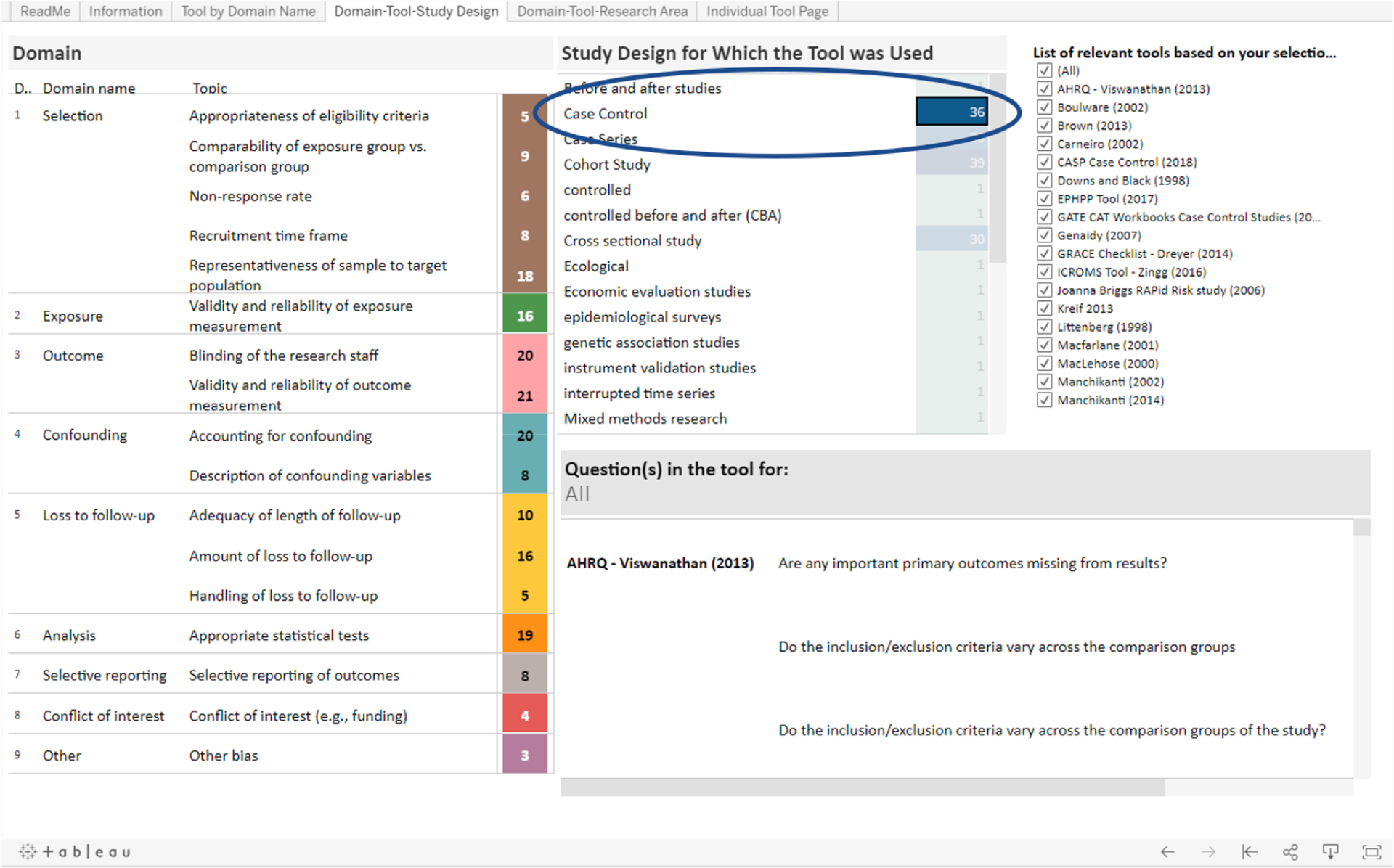
The Domain-Tool-Study Design tab allows the user to filter by the study design for which tools were developed (if applicable).

**Fig. 3b.**
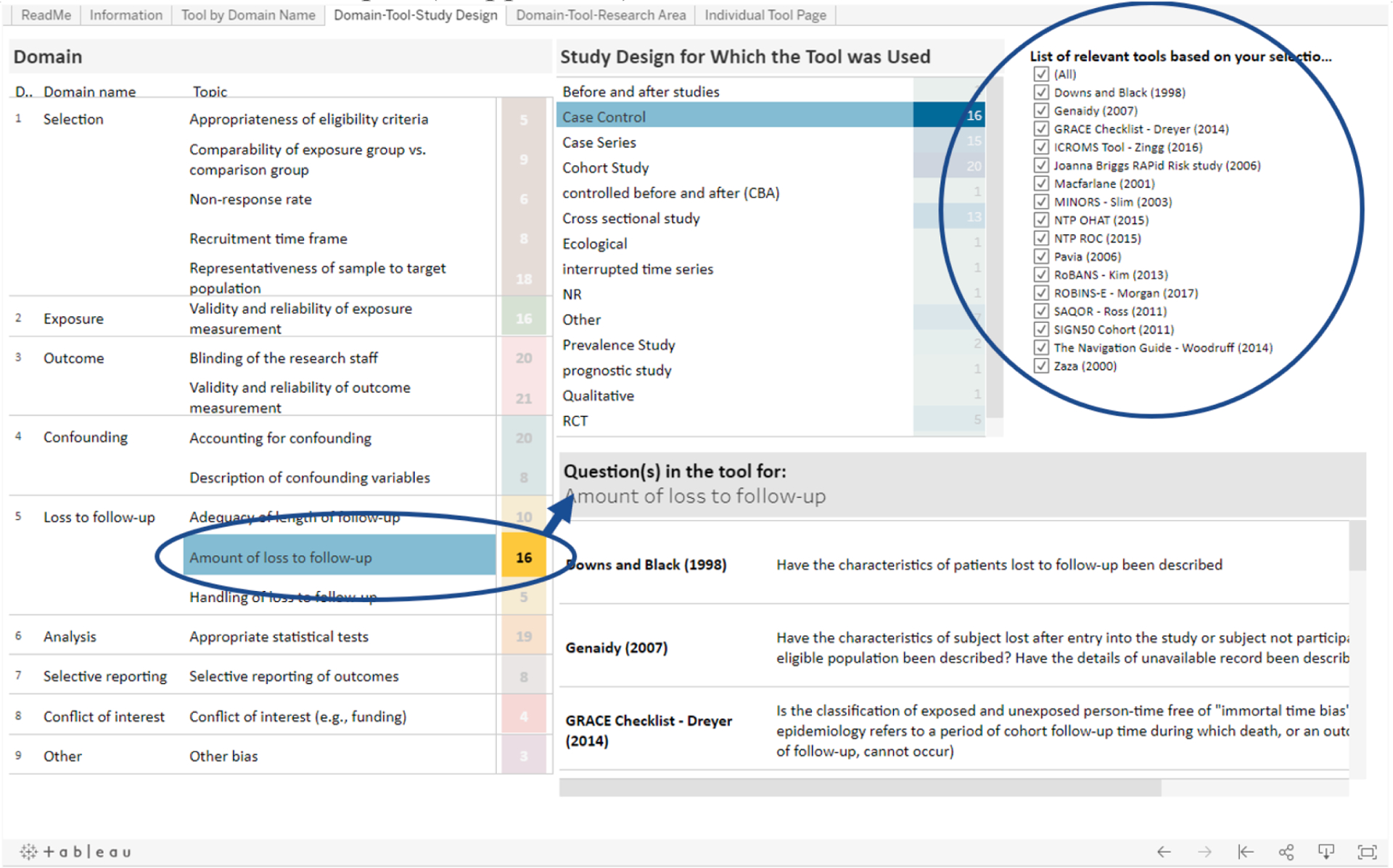
The Domain table in the Domain-Tool-Study Design tab allows the user to filter by a specific risk of bias domain; the Questions table allows the user to directly compare questions that correspond to that domain from each tool.

**Fig. 4a.**
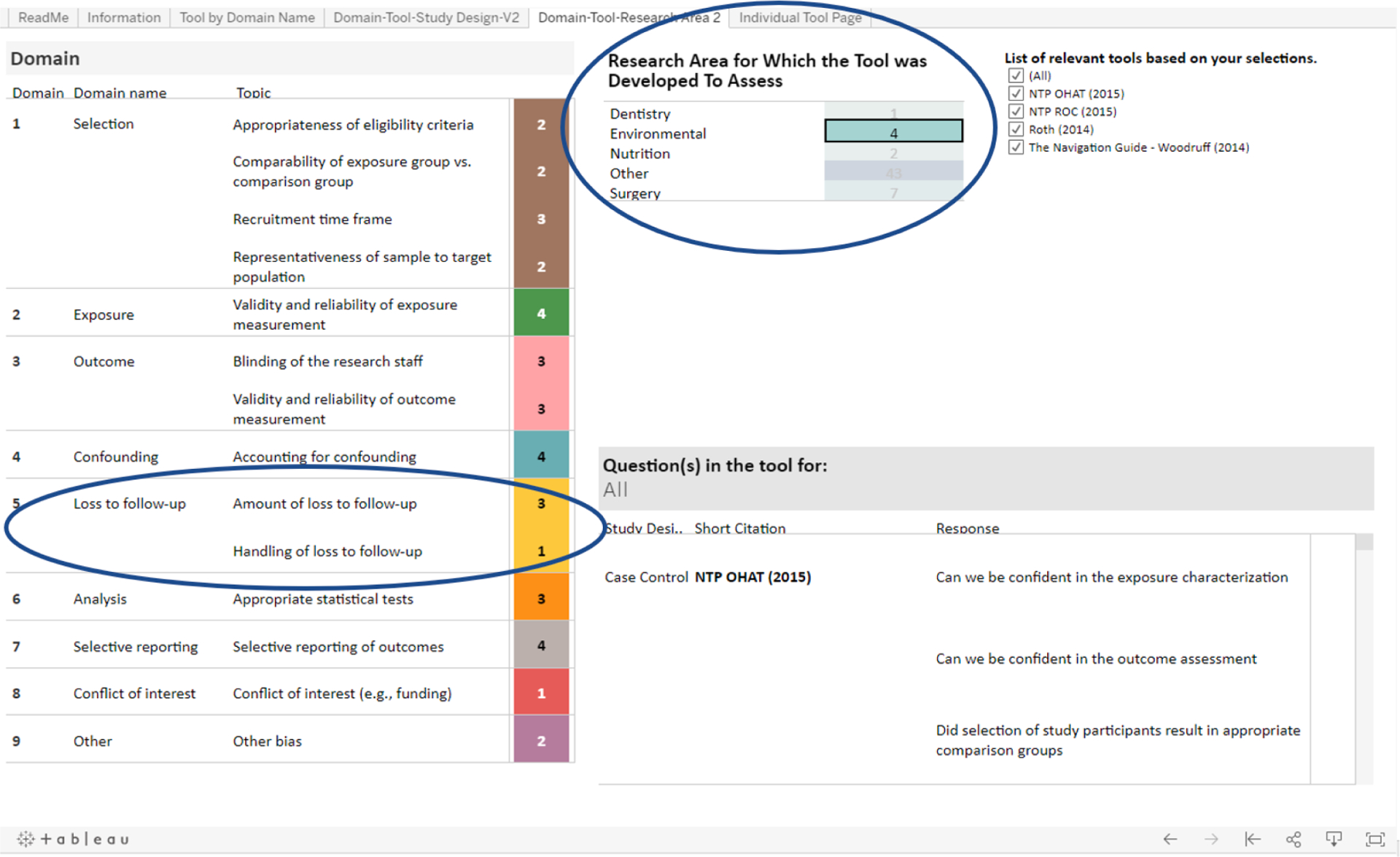
The Domain-Tool-Research Area tab allows the user to filter by the research area for which each tool was developed (if applicable).

**Fig. 4b.**
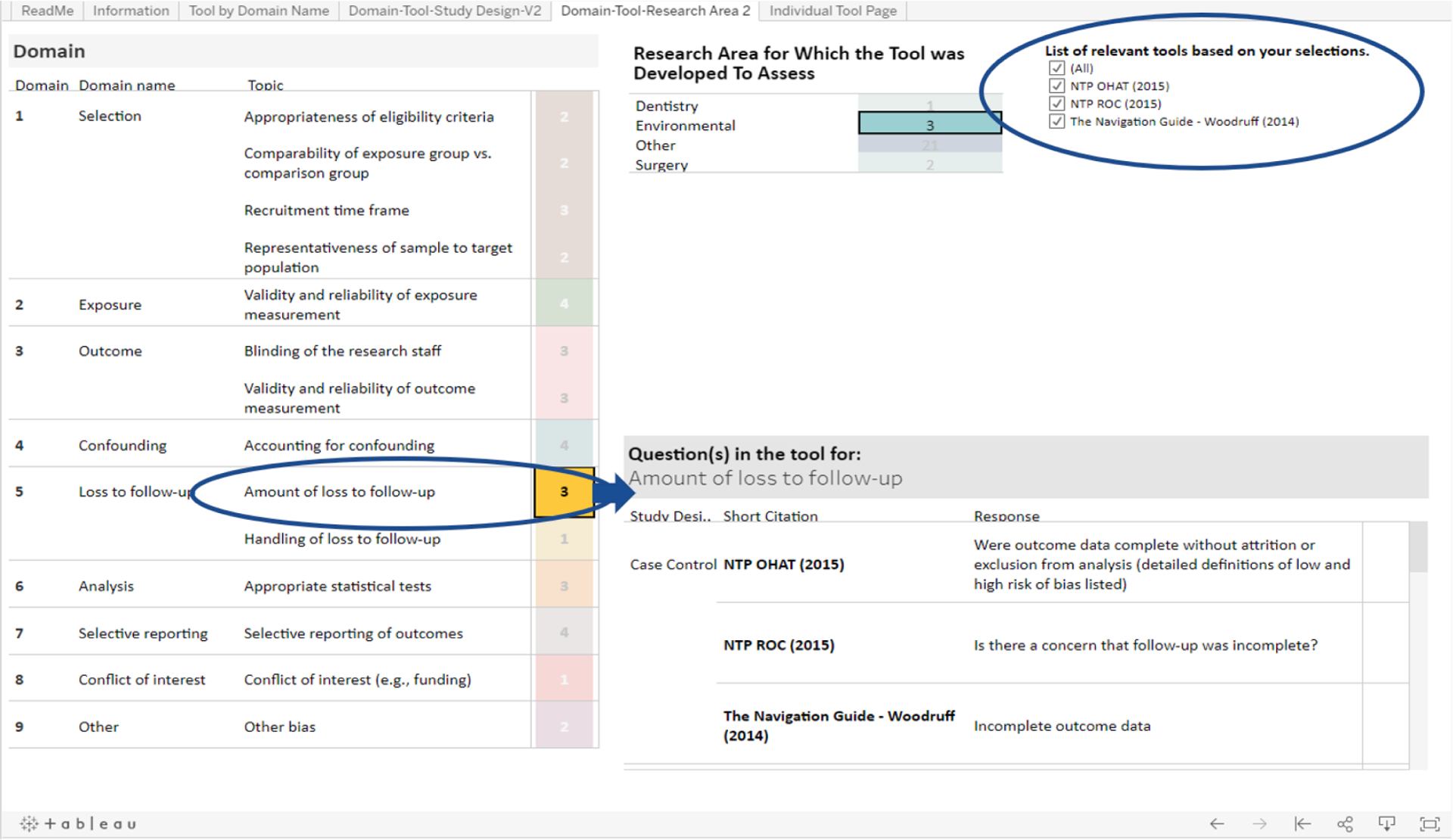
The Domain table in the Tools by Research Area tab allows the user to filter by a specific risk of bias domain; the Questions table allows the user to directly compare questions that correspond to that domain from each tool.

**Table 1:** Summary of information provided in the *Tableau* risk of bias tool finder.

Tab name in Tableau	Information gathered	Type of data
Information	Number of questions in each tool	Range: 5–53
	Number of domains in each tool	Range: 0–7 or NR
	If creators of the tool said it was tested	Yes/No/NR
	If creators of the tools tested for validity	Yes/No/NR
	If creators of tool tested for reliability	Yes/No/NR
	If the tool gives an overall quality rating	Yes/No/NR
	If the tool gives an overall quality score	Yes/No/NR
	If the tool was sponsored	Yes/No/NR
	If developers of the tool declared a COI	Yes/No/NR
Tool by Domain Name	Domain name	9 domains
	Topic	17 topics*
	Number of tools that addressed each topic	Range: 3–28
Domain-Tool Study Design	Domain name	9 domains
	Topic	17 topics
	Number of tools that addressed each topic	Range: 3–28
	Study design for which the tool was developed to assess	29 study designs
Domain-Tool-Research Area	Research area for which the tool was used	5 research areas
Individual Tool Page	Domains and questions from all available tools	Individual questions

NR: Not reported.

COI: Conflict of interest.

Table based on Wang et al. (2018).

* The individual questions from each tool that addressed the topic are also available.

## Box 1 Definitions.

**Study quality:** measure of study characteristics, including but not limited to, whether it was peer-reviewed, characteristics pertaining to internal or external validity, completeness of study reporting, best research practices (e.g., ethical approval) and / or risk of bias.

**Risk of bias:** characteristics of the design, conduct or analysis of a study related to systematic error, or deviation from the truth, in results that assess the risk of a study over- or underestimating outcome effects; this term is also used interchangeably with “internal validity”.

**Internal validity:** the validity of the inferences drawn as they pertain to the members of the source population; this term is also used interchangeably with “risk of bias” as the extent to which issues in methodological quality are likely to have prevented bias.

**External validity:** the validity of the inferences as they pertain to people outside the source population; the terms applicability, generalizability and relevance are also used for this concept.

**Reporting quality:** measure describing how clearly and completely the details and procedures of a study are described (e.g., whether the objective of the study clearly stated).
